# Fast efficient computation of expected value of sample information from a probabilistic sensitivity analysis sample: a non-parametric regression approach

**DOI:** 10.1186/1745-6215-14-S1-O25

**Published:** 2013-11-29

**Authors:** Mark Strong, Alan Brennan, Jeremy Oakley

**Affiliations:** 1 University of Sheffield, Sheffield, UK

## 

Health economic models are used to estimate the expected net benefits of competing decision options. The true values of the parameters of such models are rarely known with certainty, and it is often useful to quantify the value of undertaking further data collection (e.g. a future trial) in order to reduce uncertainty. The value of a proposed future trial can be quantified by its Expected Value of Sample Information (EVSI).

The standard approach to computing EVSI is via a nested two-level Monte Carlo sampling scheme that typically requires a large number of economic model runs. This is problematic for complex models, particularly those that require for each model run a large number of patient-level simulation steps. An additional problem arises if the EVSI inner loop requires MCMC (i.e. in those cases where the parameter distribution is not conjugate to the likelihood of the simulated trial data). In practice, these difficulties have resulted in the restriction of EVSI analyses to only a small number of published examples.

To overcome the problems above we present novel, fast and efficient non-parametric regression based method for computing EVSI. The method requires only the "probabilistic sensitivity analysis" (PSA) sample: a single set of samples from the model inputs, along with the corresponding set of model evaluations. The new method allows EVSI to be computed for a model of any complexity, and hence be made more widely available to trial designers and decision makers.

We present the method and illustrate its application in a case study.

